# ON 01910.Na (rigosertib) inhibits PI3K/Akt pathway and activates oxidative stress signals in head and neck cancer cell lines

**DOI:** 10.18632/oncotarget.12692

**Published:** 2016-10-15

**Authors:** Anil Prasad, Nagina Khudaynazar, Ramana V. Tantravahi, Amanda M. Gillum, Benjamin S. Hoffman

**Affiliations:** ^1^ Division of Experimental Medicine, Beth Israel Deaconess Medical Center, Harvard Medical School, Boston, MA 02115, USA; ^2^ Onconova Therapeutics, Inc., Newtown, PA 18940, USA

**Keywords:** rigosertib, HPV, PI3K, oxidative stress, combination therapy

## Abstract

Squamous cell carcinoma of the head and neck (HNSCC) is characterized by high morbidity and mortality. Treatment failure, drug resistance and chemoradiation toxicity have necessitated the development of alternative treatment strategies. Styryl benzyl sulfones, a family of novel small molecule inhibitors, are being evaluated as anti-neoplastic agents in multiple clinical trials. The activity of these compounds has been well characterized in several preclinical tumor studies, but their activity has yet to be fully examined in HNSCC. We tested ON 01910.Na (rigosertib), a styryl benzyl sulfone in late-stage development, in HNSCC preclinical models. Rigosertib induced cytotoxicity in both HPV(+) and HPV(−) HNSCC cells in a dose-dependent manner. Characterization of the underlying molecular mechanism indicated that rigosertib induced inhibition of the PI3K/Akt/mTOR pathway, induced oxidative stress resulting in increased generation of reactive oxygen species (ROS), and activated extracellular signal-regulated kinases (ERK1/2) and c-Jun NH2-terminal kinase (JNK). Increased phosphorylation and cytoplasmic translocation of ATF-2 were also observed following rigosertib treatment. These changes in cell signaling led us to consider combining rigosertib with HNSCC standard-of-care therapies, such as cisplatin and radiation. Our study highlights the promising preclinical activity of rigosertib in HNSCC irrespective of HPV status and provides a molecular basis for rigosertib in combination with standard of care agents for HNSCC.

## INTRODUCTION

Head and neck cancer is a major global health problem [1], and squamous cell carcinoma (HNSCC) is its most common subtype [2, 3]. Fifty percent of HNSCC patients present with regionally advanced disease [2, 4]. Despite recent progress, only 40 – 50% of these individuals survive five years post-diagnosis, and median survival for recurrent or metastatic HNSCC is less than one year [2, 4]. Many factors contribute to the etiopathogenesis of head and neck cancer [5]. Tobacco use and alcohol consumption are major risk factors [6–8], while other factors such as poor diet and dental hygiene, gastroesophageal reflux, immunosuppression, marijuana use, and various inherited syndromes may also play roles in disease pathogenesis [9]. The most well defined etiological factor for HNSCC is human papillomavirus (HPV) infection, which is associated with the fastest growing subgroup, oropharyngeal squamous cell carcinoma (OPSCC) [4, 10]. Conventional treatments for HPV(+) and HPV(−) HNSCC are often accompanied by adverse effects, and acquired resistance is common [5, 11, 12]. Thus, there is a critical need for new treatment options with enhanced safety profiles and varied mechanisms of action.

**Figure 1:**
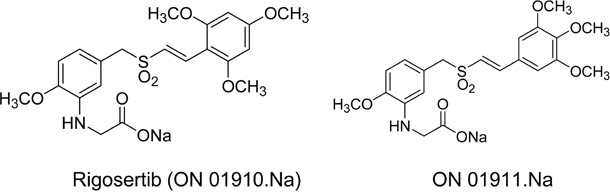
Molecular structures of rigosertib and ON 01911.Na Chemical structure of the compounds used in the study. Rigosertib (ON 01910.Na), Sodium (E)-2-(2-methoxy-5-((2,4,6-trimethoxystyrylsulfonyl)methyl)phenylamino)acetate and ON 01911.Na is an inactive isomer used as a control.

Styryl benzyl sulfones are currently being evaluated in clinical trials as potential cancer therapeutics [13–16]. These compounds are potent antimitotic agents that preferentially induce G_2_/M cell cycle arrest and apoptosis in tumor cells, with little or no effect on normal cells [14, 17, 18]. We have previously shown that one such compound, ON 01910.Na (rigosertib, Figure 1), preferentially inhibited PI3K and other oncogenic signaling cascades in mantle cell lymphoma (MCL) cell lines [19]. Here, we examined the efficacy of ON 01910.Na and a non-bioactive isomer, ON 01911 (Figure 1) on cultured HPV(−) and HPV(+) cell lines. We observed inhibition of the PI3K/Akt/mTOR pathway and induction of oxidative stress following rigosertib treatment. We also observed cytoplasmic translocation of ATF-2 in rigosertib-mediated cell death. Cytoplasmic accumulation of ATF-2 was found to sensitize tumor cells to standard-of-care HNSCC treatments, such as cisplatin and radiation. These findings suggest that rigosertib, in combination with standard-of-care therapies, could be of therapeutic value in HNSCC irrespective of HPV status, and should be studied further.

## RESULTS

### Evaluating HPV status of HNSCC cell lines

**Figure 2:**
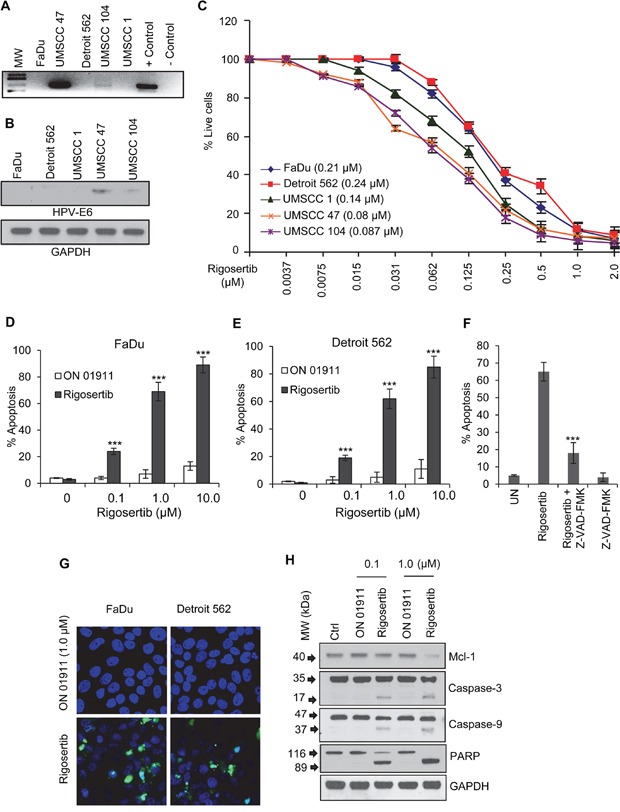
Rigosertib reduces viability and enhances apoptosis in HNSCC cell lines **A.** Evaluating HPV status in HNSCC cell lines by PCR. Total DNA from each cell line was amplified with primers to the HPV-16 *E6* gene and run on a DNA gel. **B.** Representative Western blot image of HPV E6 analysis in HNSCC cancer cell lines. GAPDH used as loading control. **C.** Cell viability as measured by MTS. FaDu, Detroit 562, UMSCC 1, UMSCC 47 and UMSCC 104 cells were treated with increasing concentrations of rigosertib for 48 h, and cell viability was assessed. 50% growth inhibition (IC_50_) is recorded for each cell line in μM in the legend. Untreated cells were considered 100% viable and percent viability of cells treated with rigosertib was calculated vs. this control. Data represent the mean +/− SD of 3 independent experiments. **D** and **E.** Apoptosis as measured by DNA fragmentation (TUNEL). FaDu and Detroit 562 cells were treated with DMSO (Ctrl), ON 01911.Na (inactive control compound) and increasing concentrations of rigosertib for 48 h. Apoptosis was assessed by TUNEL and flow cytometry and data displayed as number of apoptotic cells/total cells. Data represent the mean +/− SD of 3 independent experiments (***p ≤ 0.001). **F.** FaDu cells were treated with DMSO (UN) or rigosertib with or without Z-VAD-FMK for 48 h. Apoptosis was assessed by TUNEL and flow cytometry and data displayed as number of apoptotic cells/total cells. Data represent the mean +/− SD of 3 independent experiments (***p ≤ 0.001). **G.** Representative fluorescent microscopic images of TUNEL assay. FaDu and Detroit 562 cells incubated with 1.0 μM ON 01911.Na (control compound) or 1.0 μM rigosertib for 48 h. before fixing, performing TUNEL assay, and capturing images. Blue = DAPI. Green = Fragmented DNA. **H.** Representative Western blot analysis of the effects of rigosertib on apoptotic protein cleavage. FaDu cells were incubated with DMSO (Ctrl), or increasing concentrations of ON 01911.Na (control compound) or rigosertib for 24 h before Western blot evaluation of PARP, caspase-3, and caspase-9 cleavage, and Mcl-1. GAPDH used as loading control.

Characteristically, HPV(+) and HPV(−) HNSCCs use different molecular mechanisms to inhibit apoptosis and enhance proliferation [20], and thus respond differently to standard chemotherapeutic agents [21–25]. Therefore, we evaluated the HPV status of five HNSCC cell lines by PCR, using primers for HPV *E6*. HPV *E6* sequences were not detected in FaDu, Detroit 562, or UMSCC 1 cell lines (Figure 2A). Strong *E6* amplification was observed in UMSCC 47 cells, and weak *E6* amplification was detected in UMSCC 104 cells (Figure 2A). Further we confirmed the expression of HPV E6 protein in these two cell lines by Western blot analysis (Figure 2B).

### Rigosertib reduces viability and enhances apoptosis in HNSCC cell lines

We analyzed the effects of rigosertib on viability of five HNSCC cell lines and calculated IC_50_ values using CalcuSyn software. Cells were treated with rigosertib (0 to 2.0 μM) for 48 hours. Rigosertib significantly decreased the viability of all cell lines in a dose-dependent manner (Figure 2C). IC_50_ values were submicromolar for all cell lines tested: FaDu (0.213 μM), Detroit 562 (0.248 μM), UMSCC 1 (0.146 μM), UMSCC 47 (0.083 μM), and UMSCC 104 (0.087 μM). These data indicate that rigosertib reduced the viability of HNSCC cells, regardless of HPV status. To further characterize the mechanism by which rigosertib is cytotoxic to HNSCC cells, we incubated FaDu and Detroit 562 cell lines with 0 – 10.0 μM of rigosertib or ON 01911.Na, or DMSO for 48 hours and evaluated apoptosis using a TUNEL kit. Rigosertib induced apoptosis in a dose-dependent manner (Figure 2D & 2E). Next, we analyzed rigosertib-mediated apoptosis in the presence and absence of the pan-caspase inhibitor Z-VAD-FMK. We found that Z-VAD-FMK significantly abrogated rigosertib-mediated apoptosis in HNSCC cells indicating rigosertib induced apoptosis by activating the caspase cascade (Figure 2F). Further, fluorescence microscopic analysis of TUNEL staining revealed increased Fluorescein-dUTP labeling in rigosertib treated cells, and thus confirmed that apoptosis occurs in HNSCC cells upon rigosertib treatment (Figure 2G). To further characterize the apoptotic mechanisms, we evaluated the effects of rigosertib on PARP, Caspase-3 and Caspase-9 cleavage by Western blot analysis (Figure 2H). Cleavage of all three proteins occurred in a dose-dependent manner. Notably, we also observed decreased levels of Mcl-1, an important pro-survival protein in rigosertib treated HNSCC cells (Figure 2H). This finding is similar to our previous observations following rigosertib treatment of hematologic cancer cell lines [19, 26] and suggests that inhibition of Mcl-1 is a central mechanism by which rigosertib activates programmed cell death in cancer cells.

### Rigosertib inhibits the PI3K/Akt/mTOR signaling pathway in HNSCC cell lines

**Figure 3:**
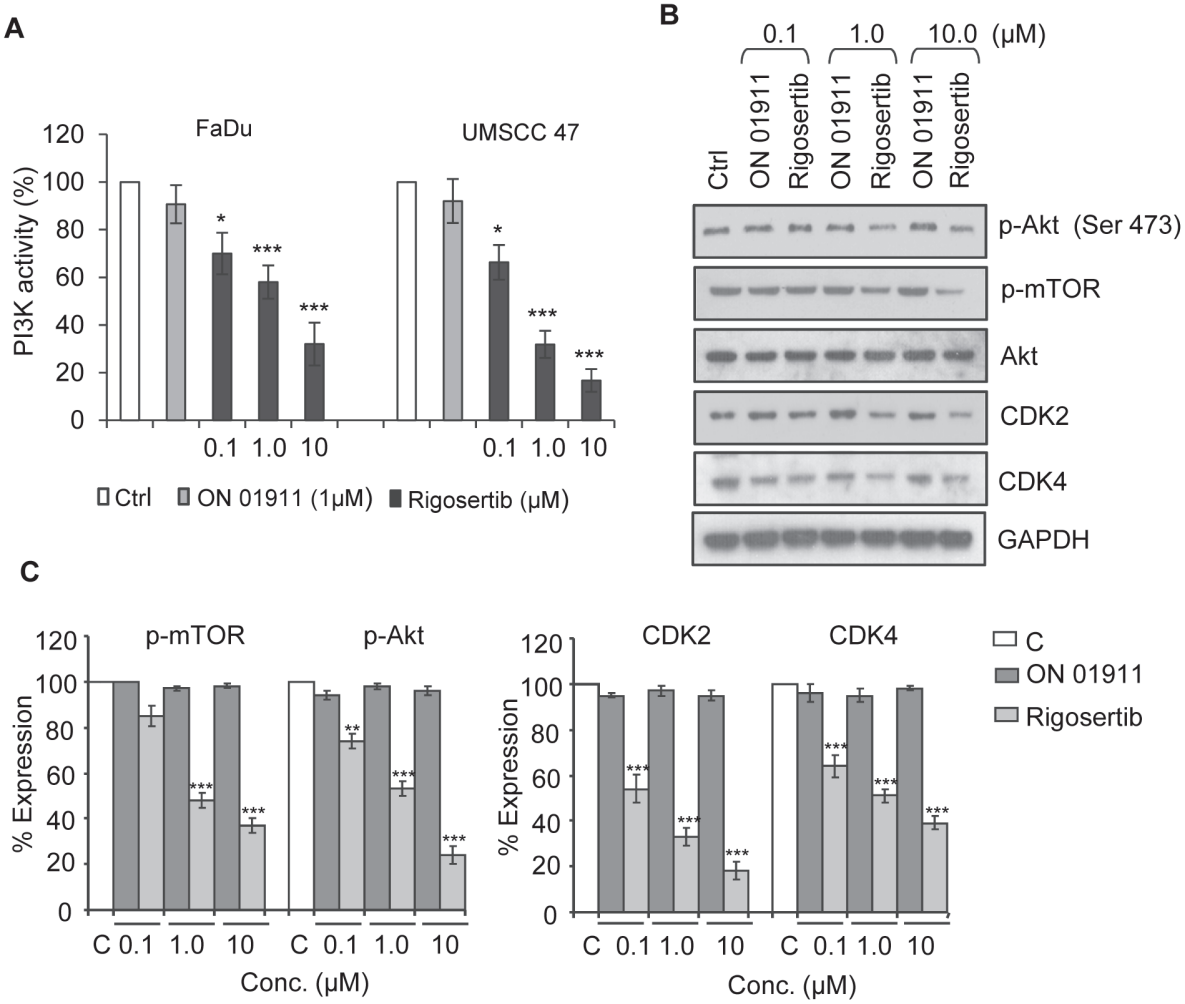
Rigosertib blocks PI3K/Akt/mTOR signaling pathway in HNSCC cell lines **A.** PI3K activity as measured by ELISA. FaDu and UMSCC 47 cells were incubated with DMSO (Ctrl), 1.0 μM ON 01911.Na (control compound), or increasing concentrations of rigosertib for 12 h. Total cell lysates were immunoprecipitated with an anti-p85 antibody, and PI3 kinase activity was assessed by ELISA. DMSO (Ctrl)-treated cells were considered to have 100% PI3K activity, and percent activity of cells treated with drugs was calculated vs. this control. Data represent the mean +/− SD of 3 independent experiments (*p ≤ 0.05; ***p ≤ 0.001). **B.** Representative Western blot analysis of the effects of rigosertib on PI3K/Akt/mTOR pathway signaling molecules. FaDu cells were incubated with DMSO (Ctrl) or with increasing concentrations of ON 01911.Na (control compound) or rigosertib for 24 h before assessing protein expression. GAPDH used as loading control. **C.** Quantitative analysis of p-mTOR, p-Akt, Cyclin D1, CDK2 and CDK4 expression in ON 01911.Na- or rigosertib-treated cells. The band intensity of each lane was determined by densitometry. Band intensity of control-treated cells was considered as 100% and all other band intensities were calculated relative to this control. Data represent the mean ± SD of 3 independent experiments (**p ≤ 0.01; ***p ≤ 0.001).

The PI3K/Akt/mTOR pathway plays a key role in the tumorigenesis of many cancers, including HNSCC [27, 28]. In previous studies, our group and others [13, 19, 26] demonstrated that rigosertib inhibits PI3K/Akt/mTOR signaling. To evaluate the effects of rigosertib on PI3K/Akt/mTOR signaling in HNSCC, we incubated FaDu and UMSCC 47 cells with DMSO (vehicle control), 1.0 μM ON 01911.Na (inactive control compound), or rigosertib (0 – 10 μM) for 12 hours, and assessed PI3K activity by ELISA. Rigosertib inhibited cellular PI3K activity in a dose-dependent manner (Figure 3A) independent of HPV status. We next examined the phosphorylation status of key components and targets of the PI3K/Akt/mTOR pathway. Western blot analysis (Figures 3B and 3C) showed that rigosertib inhibited phosphorylation of mTOR and Akt in FaDu cells. Previous studies have shown that rigosertib alters the expression of Cyclin D1 and cyclin dependent kinases (CDKs) and thereby regulates the cell cycle [18, 19]. Hence, we analyzed the expression of Cyclin D1, CDK2 and CDK4 in rigosertib-treated HNSCC cells and found that rigosertib significantly reduced the expression of these molecules in a dose-dependent manner. Taken together, these data suggest that rigosertib inhibits PI3K/Akt/mTOR signaling, resulting in inhibition of cell cycle progression.

### Rigosertib induces apoptosis in HNSCC cells by generating reactive oxygen species (ROS), which stimulates JNK pathway signaling

**Figure 4:**
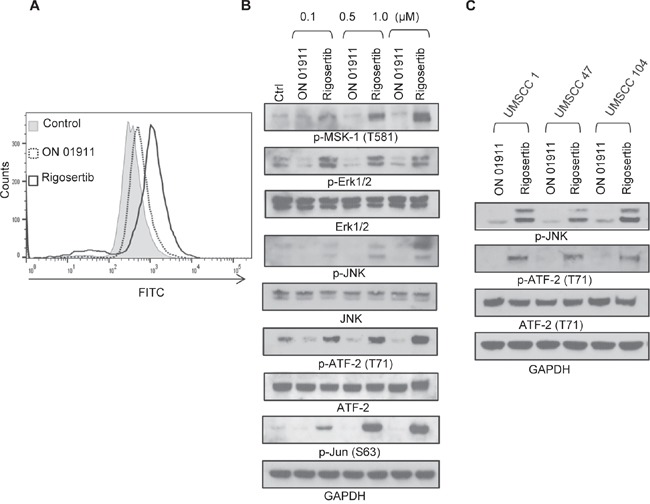
Rigosertib induces ROS generation and activates JNK pathway signaling in HNSCC cell lines **A.** Histogram plot of ROS levels as measured by flow cytometry. FaDu cells were incubated with 10.0 μM DCF-DA for 30 min, washed, then incubated with DMSO (Ctrl), 1.0 μM ON 01911.Na (control compound), or 0.1 to 10.0 μM rigosertib for 6 h before assessing ROS levels by flow cytometry. Data represent of 3 independent experiments. **B.** Representative Western blot analysis of the effects of rigosertib on JNK pathway signaling proteins. FaDu cells were incubated with DMSO (Ctrl), or increasing concentrations of ON 01911.Na (control compound) or rigosertib for 24 h before protein evaluation. GAPDH used as loading control. **C.** Representative Western blot analysis of the effects of rigosertib on the phosphorylation of JNK and ATF2 and total ATF2 in multiple HNSCC cell lines. UMSCC 1, UMSCC 47 and UMSCC 104 cells were incubated with 1.0 μM ON 01911.Na (control compound) or 1.0 μM rigosertib for 24 h before protein evaluation. GAPDH used as loading control.

Previous studies in non-HNSCC tumors demonstrated that rigosertib stimulates reactive oxygen species (ROS) generation and JNK pathway activation [19, 26]. We evaluated the effect of rigosertib on these pathways in HNSCC cell lines. Rigosertib treatment led to dose-dependent increases in ROS in FaDu cells (Figure 4A). Since ROS generation is known to activate cellular stress pathways via JNK and p38 and to trigger apoptosis [29], we extended our studies to evaluate the activation of JNK/AP-1 pathway proteins. MSK1 is a stress-activated protein kinase that is activated downstream of both the ERK1/2 and p38 MAPK cascades [30]. We observed rigosertib-enhanced phosphorylation of Erk1/2 and MSK-1 in a dose-dependent manner, thus indicating activation of this stress response pathway (Figure 4B). Our analysis of the JNK pathway showed significant increases in phosphorylated JNK, c-Jun and ATF2 following rigosertib treatment. Activated JNK promotes apoptosis by stimulating c-Jun/AP1 responsive gene expression and by ATF2 translocation to the mitochondria and cytoplasmic release of cytochrome c [29]. To determine whether rigosertib activates JNK-dependent apoptosis in both HPV(+) and HPV(−) cell lines, we incubated UMSCC 1 (HPV-), UMSCC 47 (HPV+), and UMSCC 104 (HPV+) cells with 1.0 μM ON 01911.Na or 1.0 μM rigosertib for 24 hours and then assessed the activation state of JNK and ATF-2 by Western blot analysis (Figure 4C). Rigosertib enhanced the phosphorylation of JNK and ATF-2 in all three HNSCC cell lines examined, indicating activation of cellular stress response through JNK signaling in both HPV(+) and HPV(−) cell lines.

### Rigosertib induces ATF-2 translocation to the mitochondria

**Figure 5:**
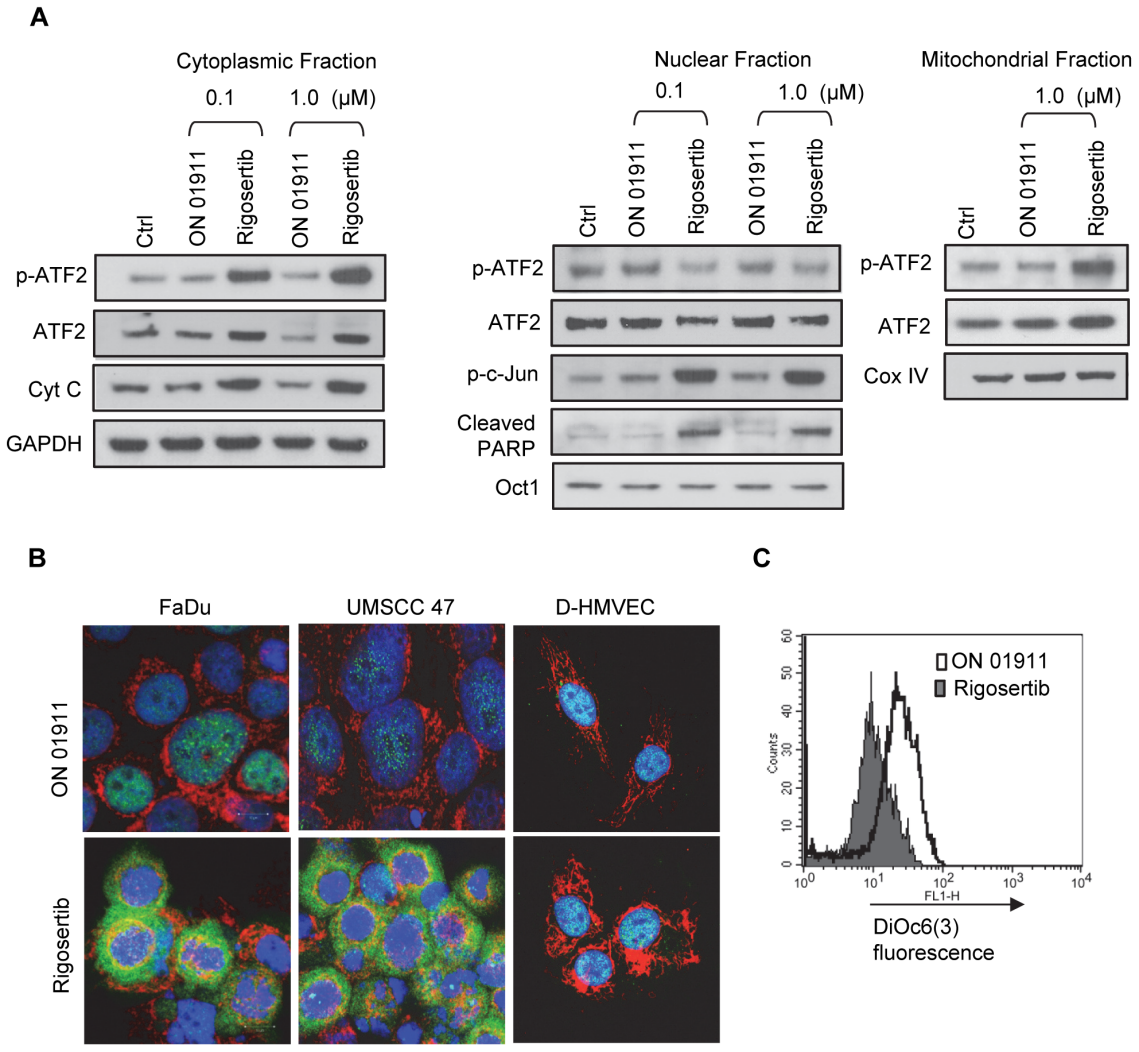
Rigosertib induces translocation of ATF2 from the nucleus to the cytoplasm, and its co-localization to mitochondria in HNSCC cell lines **A.** The effects of rigosertib on the subcellular localization of ATF2 in HNSCC cells. FaDu cells were treated with DMSO (Ctrl) and various concentrations of rigosertib or ON 01911.Na (control compound) as indicated for 24 h before separating the lysates into cytoplasmic, nuclear, and mitochondrial fractions. Subcellular fractions were examined by Western blot analysis using indicated antibodies. GAPDH used as loading control for cytoplasmic fraction. Oct1 used as purity and loading controls for nuclear fractions. Cox IV used as purity and loading controls for mitochondrial fractions. **B.** Representative confocal microscopy images of FaDu, UMSCC 47 and D-HMVEC cells treated with rigosertib (1.0 μM) or ON 01911.Na (1.0 μM) for 24 h before fixing, staining with indicated antibodies, and capturing images. Green: p-ATF; Red: Cox IV (mitochondrial marker); Blue: DAPI (nuclear marker). **C.** The effects of rigosertib on mitochondrial transmembrane potential. FaDu cells were treated with DMSO (Ctrl), 1.0 μM rigosertib, or 1.0 μM ON 01911.Na (control compound) for 24 h, then incubated in the dark with DiOC6(3) [40 nM] for 15 min at 37°C, and analyzed by flow cytometry. Filled histogram represents rigosertib-treated cells. Open histogram represents ON 01911.Na-treated cells.

Rigosertib treatment leads to a dramatic increase in phosphorylated ATF-2, a stress-associated kinase associated with many critical cellular processes [31]. Previous studies have suggested a role for ATF-2 in oncogenic and tumor-suppressor functions [32]. A recent study by Varsano et al. [33] has shown that translocation of ATF-2 from the nucleus to the mitochondrial outer membrane, inhibits the growth of melanoma cells and induces apoptosis. Hence, we sought to further analyze the role of ATF-2 in rigosertib-mediated effects in HNSCC. To that end, we separated subcellular fractions from vehicle, ON 01911.Na-, and rigosertib-treated FaDu cells and analyzed the levels of total and p-ATF-2. We observed dramatic increase in p-ATF-2 and ATF-2 levels in both cytoplasmic and mitochondrial fractions following rigosertib treatment suggesting nuclear export (Figure 5A). Rigosertib-dependent translocation of p-ATF-2 occurred in HNSCC independent of HPV status, but did not occur in Normal Human Dermal Microvascular Endothelial Cells (HMVEC-d), suggesting that rigosertib-induced translocation of ATF-2 may be specific to tumor cells (Figure 5B). Concomitant with ATF-2 translocation, we observed higher levels of cytochrome c (Cyt c) in the cytoplasmic fraction and increased cleaved PARP levels in the nuclear fraction of HNSCC cells treated with rigosertib (Figure 5A). Since ATF-2 nuclear export and translocation to the mitochondria is known to perturb the HK1-VDAC1 complex and to increase mitochondrial permeability [34, 35], we analyzed mitochondrial membrane potential using DiOC6(3) pre-loaded FaDu cells. Treatment with rigosertib resulted in decreased fluorescence, indicating that drug treatment altered mitochondrial membrane potential, which may have induced apoptosis of these cells (Figure 5C).

### Rigosertib in combination with chemotherapy or radiation induces significant cytotoxicity in HPV cell lines

**Figure 6:**
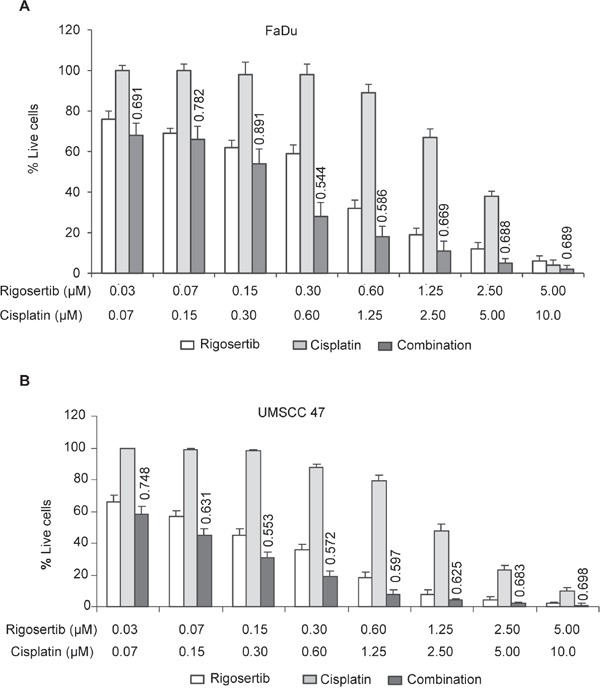
Combined treatment of rigosertib and cisplatin induces significant cytotoxicity in HPV(−) and HPV(+) cell lines Cell viability as assessed by MTS. FaDu **A.** and UMSCC 47 cells **B.** were incubated with rigosertib alone [0.03 μM – 5.0 μM], cisplatin alone [0.07 μM – 10.0 μM], or a combination of both drugs for 48 h before calculating viability. Combinational index (CI) values were calculated with CalcuSyn software, and their values displayed above “combination” treatment columns. Concentrations at which synergism was detected are indicated (< 0.9 CI = synergism; 0.9-1.0 CI = additive). Data represent the mean +/− SD of 3 independent experiments.

Cytoplasmic accumulation of ATF-2 resulting in altered mitochondrial membrane potential has been shown to enhance the sensitivity of cancer cells to cytotoxic agents [33, 36, 37] and radiation [38]. Rigosertib, through activation of the JNK pathway and ATF-2 translocation, could serve as a sensitizing agent for conventional cytotoxic therapy and radiation. To test this hypothesis, we analyzed the combination of rigosertib and cisplatin, a standard-of-care cytotoxic agent for HNSCC (Figures 6A and 6B). FaDu and UMSCC 47 cell lines were incubated with rigosertib (0.03 μM – 5.0 μM), cisplatin (0.07 μM – 10.0 μM), or a combination of the two drugs for 48 hours. Cellular viability was calculated by MTS (Cell Titer 96^®^ Aqueous Non-Radioactive Cell Proliferation Assay) and combination index (CI) values were determined using CalcuSyn software. The combination of rigosertib and cisplatin displayed additivity or synergism (i.e., CI < 1) at multiple concentrations, indicating that the administration of both drugs has a greater effect on HNSCC cell viability than either drug alone (Figures 6A and 6B).

**Figure 7:**
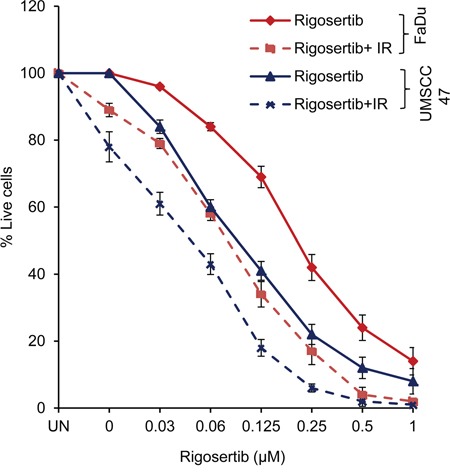
Rigosertib radiosensitizes both HPV (−) and HPV (+) HNSCC cell lines FaDu and UMSCC 47 cells were pretreated with increasing concentrations (0-1μM) of rigosertib for 24 h, then cells exposed to radiation (6Gy). After 24 h, cell viability was measured by using Cellometer AutoT4 cell counter. Untreated cells were considered 100% viable and percent viability of each sample was calculated. Data represent the mean +/− SD of 3 independent experiments.

Recent preclinical studies by Agony et al [39] have shown that rigosertib is a more effective radiosensitizer than cisplatin in concurrent chemoradiation treatment of cervical carcinoma both *in vitro* and *in vivo*. Expanding on these findings, we hypothesized that the effect of rigosertib on ATF-2 and mitochondrial membrane potential would likely sensitize HNSCC cells to radiation treatment. To this end, we analyzed the combinational effects of rigosertib and radiation in HPV(−) FaDu cells and HPV(+) UMSCC 47 cells. Cell lines were pretreated with various concentrations (0 - 1.0 μM) of rigosertib for 24 hours and then subjected to radiation (6Gy). Cytotoxicity was measured 24 hours after irradiation. Cell death increased significantly with combination treatment compared to treatment with rigosertib or radiation alone (Figure 7). The combination of rigosertib and radiation was active in both HPV(+) and HPV(−) cell lines, although we observed slightly greater efficacy in HPV(+) cells.

## DISCUSSION

Squamous cell carcinoma of the head and neck is a common cancer with high morbidity and limited therapeutic options [2, 4, 5]. The small molecule inhibitor, rigosertib, was previously shown to be an effective anti-proliferative agent in HNSCC cell lines and HNSCC patient-derived xenografts [40]. Here, we studied the effects of rigosertib in HPV(−) and HPV(+) HNSCC cell lines and characterized combination approaches of this novel anti-cancer agent with standard-of-care therapies.

In previous studies, we and others have shown that rigosertib inhibits the PI3K pathway [19, 26, 40]. Activation of PI3K and its role in oncogenesis, progression and resistance to chemotherapeutic agents has become more evident in recent studies of HNSCC [27, 28, 41]. Furthermore, HPV 16 oncoproteins E6 and E7, are known to induce activation of the PI3K/Akt pathway and have been directly linked to the pathogenesis of HPV(+) HNSCC and its resistance to the standard of care agent, cisplatin [42–45]. In the current, study we observed that rigosertib inhibited cellular PI3K pathway activity in both HPV(+) and HPV(−) HSNCC cells, while also blocking the activation of key downstream effector proteins in survival and cell cycle control pathways.

Recent evidence suggests that rigosertib acts to inhibit PI3K activity through a mechanism that may affect multiple signaling cascades leading to cancer cell death. We therefore explored the effect of rigosertib on additional complementary pathways related to apoptosis in order to further understand the activity of rigosertib in HNSCC beyond PI3K inhibition. In a recent study Chapman et al [26] demonstrated ROS-induced oxidative stress responses upon treatment with rigosertib. Similarly, our analysis revealed that rigosertib treatment led to a robust increase in ROS levels and further triggered the stress response cascade by activation of MAPK pathways (ERK and JNK/SAPK) and phosphorylation and nuclear accumulation of c-JUN and ATF-2.

The dramatic increase in phosphorylated ATF-2 was notable and led us to further investigate the role of this protein in rigosertib activity. ATF-2, a member of ATF/CREB family of transcription factors, is activated by Jun N-terminal kinase (JNK), p38, or extracellular-signal-regulated kinase 1 (ERK1) through phosphorylation events at Thr69 and Thr71 [31]. Activated ATF-2 binds to the AP1 complex to regulate diverse cellular functions and can either act in tumor promotion or tumor suppression depending on its sub-cellular localization [32, 33, 46, 47]. The exact molecular mechanisms involved in this differential action have yet to be elucidated. However, recent studies suggest that cellular stress or genotoxic stimuli induce cytoplasmic translocation of ATF-2 and inhibit its transcriptional activity [33]. Once in the cytoplasm, ATF-2 localizes to the outer membrane of mitochondria, where it binds to the HK1 and VDAC1 complexes that regulate mitochondrial membrane potential and pore permeability [32, 34, 35]. Mobilization of ATF-2 to mitochondria alters the HK1-VDAC1 complex, resulting in disruption of mitochondrial membrane potential, activation of pro-apoptotic Bcl-2 family proteins and leakage of cytochrome c [32].

Our studies indicated that rigosertib treatment leads to cytoplasmic translocation of phosphorylated and total ATF-2, thus altering the mitochondrial membrane potential in HNSCC cells. Disruptions in mitochondrial membrane potential were associated with increased levels of cytoplasmic cytochrome c, indicating induction of mitochondrial-mediated apoptosis by rigosertib. We did not observe these effects in HUVEC (D-HMVEC) cells, suggesting that these rigosertib-mediated cytotoxic effects occur only in tumor cells. Taken together, these findings indicate that rigosertib induces apoptosis in HNSCC cells, independent of HPV status, by a central mechanism that involves ATF-2.

Cytoplasmic accumulation of ATF-2 and altered mitochondrial membrane potential have been shown to enhance the sensitivity of cancer cells to cytotoxic agents and radiation [33, 36–38], leading us to hypothesize that rigosertib could act synergistically in combination with HNSCC standard-of-care therapies. Treatment with rigosertib was found to significantly enhance apoptosis of HNSCC when combined with chemotherapy or radiation. Additive or synergistic activity was noted when rigosertib was combined with cisplatin or radiation, suggesting that the combination of rigosertib with cisplatin or radiation could be an effective therapeutic strategy against HNSCC.

During the preparation of this manuscript, Athuluri-Divakar et al [48] reported the molecular mechanism of action of rigosertib. Rigosertib acts as a RAS mimetic by binding directly to the Ras Binding Domains (RBDs) of RAS effector proteins. Consequently, rigosertib acts to inhibit multiple potentially oncogenic signaling pathways including those of the RAS-RAF-MEK axis. *In vitro* binding assays also indicated that rigosertib binds directly to the RBD of PI3K.

In 2006, cetuximab, a human mouse chimeric monoclonal anti-EGFR antibody was approved in conjunction with radiotherapy for locally advanced HNSCC [49]. Cetuximab treatment, while efficacious, is also associated with a resistance phenotype, thus limiting its effectiveness as an HNSCC treatment. In 2014, Rampias et al [50], using patient biopsies, cetuximab-resistant cell lines, and transgenic mice designed to form RAS-driven oral tumors determined the basis for *de novo* cetuximab resistance. Oncogenic HRAS induces tumorigenesis in HNSCC following activation of ERK signaling leading to expression of a specific gene signature involving C-myc, BCL-2, BCL-X_L_ and cyclin D1. In 2016, Braig et al [51], used liquid biopsy specimens from HNSCC patients to examine genetic changes accompanying cetuximab treatment via next generation sequencing. In a significant number of patients tested, RAS mutations were infrequent before treatment, but arose in response to cetuximab exposure. This led to the conclusion that RAS mutations provide the basis for cetuximab resistance and for HNSCC disease progression. Rigosertib acts to suppress RAS signaling through its association with the RBDs of RAS downstream effectors, and could be added to cetuximab/radiotherapy protocols to treat patients who have exhibited cetuximab resistance.

## MATERIALS AND METHODS

### Cell lines and reagents

FaDu and Detroit 562 cell lines were purchased from American Type Culture Collection (ATCC, Manassas, VA) and cultured per ATCC recommendations. HPV(−) cell line UMSCC 1 and HPV(+) cell lines UMSCC 47 and UMSCC 104 were kindly provided by the laboratory of Dr. Thomas Carey, University of Michigan. Cells were maintained at 37°C in a humidified, 5% CO_2_ atmosphere and cultured in Dulbecco's Modified Eagle's medium (DMEM), supplemented with 10% FBS, 1% penicillin and streptomycin, and MEM non-essential amino acids (Gibco™, Life Sciences Solutions Group, Thermo Fisher Scientific, Carlsbad, CA). Rigosertib (ON 01910.Na) and ON 01911.Na (an inactive control analog) (Figure 1) were provided by Onconova Therapeutics, Inc. (Newtown, PA). These compounds were dissolved in DMSO. Cisplatin was purchased from Sigma-Aldrich (St. Louis, MO). Antibodies to cleaved PARP, cleaved Caspase-3, cleaved Caspase-9, p-mTOR, p-Akt (Ser 473), Akt, p-MSK-1 (T581), p-Erk1/2, Erk1/2, JNK, p-JNK, p-ATF-2 (T71), ATF-2, p-Jun (S63) and COX IV were purchased from Cell Signaling Technology (Danvers, MA). Antibodies to Mcl-1, GAPDH, CDK2, CDK4, Cyt C, and Oct1 were purchased from Santa Cruz Biotechnology (Santa Cruz, CA).

### PCR analysis

Total DNA (100 ng) from each cell line was amplified in Bio-Rad MyCycler for 30 cycles. The amplified product was electrophoresed through 2% agarose gel. The following primers were used to amplify the viral E6 gene: HPV-16-F: ACGTTGGATGATGTTCAGGACCCACAGGA and HPV-16- R: ACGTTGGATGCACGTCGCAGAACTGTTGC. These primers were ordered from Integrated DNA technologies, (Coralville, Iowa).

### Cell viability assay

Cell viability was examined by using the Cell Proliferation Assay Kit (Promega, Madison, WI) per the manufacturer's instructions. FaDu, Detroit 562, UMSCC 1, UMSCC 47 and UMSCC 104 cells were plated at a density of 4 × 10^3^ cells per well of a 96-well plate with or without indicated concentrations of rigosertib. IC_50_ values for rigosertib on each cell line were determined using CalcuSyn software (Biosoft, Cambridge, UK).

### TUNEL assay

Terminal deoxynucleotidyl transferase dUTP nick end labeling (TUNEL) was conducted using Cell Death Detection Kit, Fluorescein (Roche Diagnostics, Basel, Switzerland). Cells were treated as described above with various concentrations of rigosertib for 48 hours and stained with TUNEL reaction mixture per the manufacturer's instructions. Staining was quantified using a FACSVANTAGE™ flow cytometer (BD Biosciences, San Jose, CA) or images were captured by fluorescent microscopy.

### Detecting reactive oxygen species

Analysis of reactive oxygen species (ROS) was performed as described previously [52]. Cells were incubated with 10 μmol/L 5-(and-6)-carboxy-2',7'-dichlorodihydrofluorescein diacetate (H_2_DCFDA) (Invitrogen™, Life Sciences Solutions Group, ThermoFisher Scientific) for 30 minutes, washed, and incubated with DMSO, 1.0 μM ON 01911.Na, or 0 – 1.0 μM rigosertib for 12 hours. ROS (fluorescent green) were quantified by flow cytometry.

### Analysis of mitochondrial membrane transition

The mitochondrial membrane potential (MMP) probe 3,3′-dihexyloxacarbocyanine iodide [DiOC6(3)] (Invitrogen™) was used to study the decrease of MMP following drug treatment via flow cytometry. Cells were treated with drug or control for 12 hours, stained with DiOC6(3) (40 nM for 20 minutes), and analyzed by flow cytometry (BD Biosciences). A total of 10,000 events were recorded for each analysis.

### Preparation of mitochondrial, nuclear and cytosolic and fractions

Subcellular fractions were prepared using the Mitochondria/Cytosol Fractionation Kit (Biovision Mountain View, CA, USA) and NE-PER™ Nuclear and Cytoplasmic Extraction Reagents (ThermoFisher Scientific) per manufacturer's instructions. A COX IV antibody was used to monitor the purity of the mitochondrial fraction.

### Western blotting

Drug-treated cells were lysed, solubilized and electrophoresed and blotted as described previously [19]. Equal amounts of cell lysates were separated and subjected to Western Blotting using the antibodies described above.

### Irradiation

Irradiation of HNSCC cells was performed as described previously [39]. Cells were pretreated with drug or control and then exposed to radiation (6Gy) using a γ-ray irradiator. Twenty-four hours later, cell viability was assessed using a Cellometer® AutoT4 Cell Viability Counter (Nexcelom Bioscience, Lawrence, MA).

### Confocal microscopy

FaDu, UMSCC 47 and D-HMVEC cells were treated with drug or control for 24 hours and then fixed 4% paraformaldehydeand blocked with 5% normal goat serum in PBS/Triton. Cells were then incubated with relevant antibodies, washed with PBS, and stained with Alexa Fluor® 568–labeled anti–mouse IgG antibody (Molecular Probes®, Life Sciences Solutions Group) and/or Alexa Fluor® 488 phalloidin (Molecular Probes®). Cells were again washed with PBS, and slides were mounted using ProLong® Gold Antifade Mountant with DAPI (4',6-diamidino-2-phenylindole; Molecular Probes®). Slides were examined under a Zeiss 510 Meta confocal microscope (Carl Zeiss Microimaging, LLC, Thornwood, NY), and images were acquired using LSM 510 software (Carl Zeiss).
